# Clinical Implications of *UGT1A1* Polymorphisms in Anticancer Therapy: An Updated Review

**DOI:** 10.3390/pharmaceutics18080928

**Published:** 2026-07-29

**Authors:** Silvia R. Vitale, Melissa Drago, Federica Martorana, Chiara Conti, Michele Massimino, Stefania Stella, Cristina Tomarchio, Paolo Vigneri, Livia Manzella

**Affiliations:** 1 Center of Experimental Oncology and Hematology, A.O.U. Policlinico “G. Rodolico-S. Marco”, 95123 Catania, Italy; silviarita.vitale@gmail.com (S.R.V.); michedot@yahoo.it (M.M.); stefania.stella@unict.it (S.S.); cristina.tomarchio@hotmail.it (C.T.); manzella@unict.it (L.M.); 2 Department of Clinical and Experimental Medicine, University of Catania, 95123 Catania, Italy; dragomelissa23@gmail.com (M.D.); conchiara9@gmail.com (C.C.); paolo.vigneri@unict.it (P.V.); 3 Humanitas Istituto Clinico Catanese, Misterbianco, 95125 Catania, Italy; 4 Department of General Surgery and Medical-Surgical Sciences, University of Catania, 95123 Catania, Italy

**Keywords:** pharmacogenetics, *UGT1A1* polymorphisms, anticancer therapy, adverse events, drug–drug interactions

## Abstract

Pharmacogenetics has transformed oncology by enabling personalized, effective and safer use of anticancer therapies. Among clinically relevant pharmacogenetic biomarkers, polymorphisms in the *UGT1A1* gene influence the metabolism of irinotecan and other SN-38-based agents, increasing the risk of treatment-related toxicities. Beyond SN-38-based therapies, *UGT1A1* contributes to the metabolism of endogenous compounds and several drugs, and its activity may be affected by concomitant inhibitors or inducers, highlighting its broader clinical relevance. Although substantial evidence supports the association between *UGT1A1* genotype and drug safety, implementation of genotype-guided treatment remains inconsistent. This narrative review summarizes the biological basis and clinical relevance of *UGT1A1* polymorphisms in patients receiving SN-38-based therapies. Original studies, systematic reviews, meta-analyses, and international and regional pharmacogenetic guidelines were evaluated regarding genotype-guided dosing, clinical outcomes, implementation strategies, and barriers to routine testing. Reduced-function *UGT1A1* variants are associated with increased risk of irinotecan-induced neutropenia and diarrhea, particularly at higher doses. However, recommendations from scientific societies differ regarding testing indications, dose adjustment strategies, and patient selection. Uncertainties remain concerning the clinical implications of *UGT1A1* variability for newer antibody–drug conjugates carrying an SN-38 payload. Practical barriers, including inconsistent guideline recommendations, limited access to testing, reimbursement issues, and heterogeneous clinical workflows, continue to restrict implementation. *UGT1A1* genotyping represents a valuable tool for improving the safety of selected anticancer therapies, yet its incorporation into clinical practice remains incomplete. As precision oncology evolves, pharmacogenetic testing may optimize treatment selection, reduce toxicity, and improve outcomes. Further harmonization of international recommendations, high-quality prospective studies, and wider access to pharmacogenetic testing are needed to support routine implementation.

## 1. Introduction

In recent decades, advances in the understanding of tumor biology have led to substantial progress in cancer therapy, as shown by the development of highly effective chemotherapy regimens and targeted agents [1]. More recently, innovative therapeutic strategies, including antibody–drug conjugates (ADCs), have further expanded the range of available anticancer treatments. As therapeutic options have expanded and patient outcomes have improved, increasing attention has been directed toward treatment-related toxicities and their impact on health-related quality of life [2]. In this context, pharmacogenetics seeks to leverage genetic testing to optimize treatment efficacy while minimizing adverse events [3].

A well-established example is the mandatory testing for dihydropyrimidine dehydrogenase (*DPD*) polymorphisms before initiating fluoropyrimidine-based therapy, as such variants impair drug metabolism and can cause severe or even fatal toxicities [4].

Similarly, polymorphisms in the *uridine diphosphate glucuronosyltransferase* 1A1 (*UGT1A1*) gene may markedly alter enzymatic activity, predisposing patients to toxicities from various anticancer drugs. Under physiological conditions, the UGT1A1 enzyme plays a central role in the glucuronidation of bilirubin, steroids, xenobiotics, and multiple drugs, facilitating their excretion in a water-soluble form [5]. More than 50 *UGT1A1* variants associated with reduced enzymatic activity have been identified, several of which underlie hereditary unconjugated hyperbilirubinemia, including Gilbert and Crigler–Najjar syndromes [5].

Beyond bilirubin metabolism, *UGT1A1* variants can increase the risk of chemotherapy-induced toxicities, either by impairing drug clearance or by enhancing susceptibility to drug–drug interactions (DDIs) [6,7]. This is particularly relevant for irinotecan and other SN-38-based therapies, in which reduced UGT1A1 activity increases exposure to the active metabolite SN-38 and may contribute to severe toxicities, including neutropenia and diarrhea.

Although previous reviews have comprehensively summarized the biological role of *UGT1A1* and its association with irinotecan toxicity, several clinically relevant questions remain unresolved regarding the implementation of genotype-guided treatment in routine oncology practice. Furthermore, many earlier reviews predate the approval of SN-38-based antibody–drug conjugates and the publication of updated international pharmacogenetic guidelines, warranting an updated synthesis of the available evidence. In particular, uncertainty persists regarding optimal testing strategies, dose-adjustment approaches, patient selection, and the clinical relevance of UGT1A1 variability in the context of emerging anticancer therapies.

Several international guidelines provide recommendations on *UGT1A1* testing; however, univocal consensus across scientific societies is lacking [8]. These discrepancies largely reflect differences in the interpretation of available clinical evidence, recommended dose thresholds, and the balance between treatment efficacy and toxicity prevention. Differences among guidelines regarding testing indications, dosing strategies, and implementation approaches continue to limit the adoption of genotype-guided treatment. Moreover, emerging SN-38-based antibody–drug conjugates, such as sacituzumab govitecan, highlight the need for updated evaluation of UGT1A1-related evidence.

We performed a narrative review of the literature, from inception to 2025, looking for original articles, systematic reviews, meta-analyses and pharmacogenetic guidelines to provide an updated overview of the biological and clinical relevance of *UGT1A1* polymorphisms, critically assess current guideline recommendations, and discuss implementation challenges and future perspectives for integrating UGT1A1 testing into precision oncology.

## 2. UGT1A1: From Gene to Protein

The mammalian UGT superfamily contains 117 members divided into four families, *UGT1*, *UGT2*, *UGT3* and *UGT8*. The complex *UGT1A* locus is situated on chromosome 2q37 and consists of a series of coding regions that enables the transcription of at least 11 functional isoforms, of which UGT1A1 is the main enzyme, belonging to the UGT1A subfamily [9,10].

**Figure 1:**
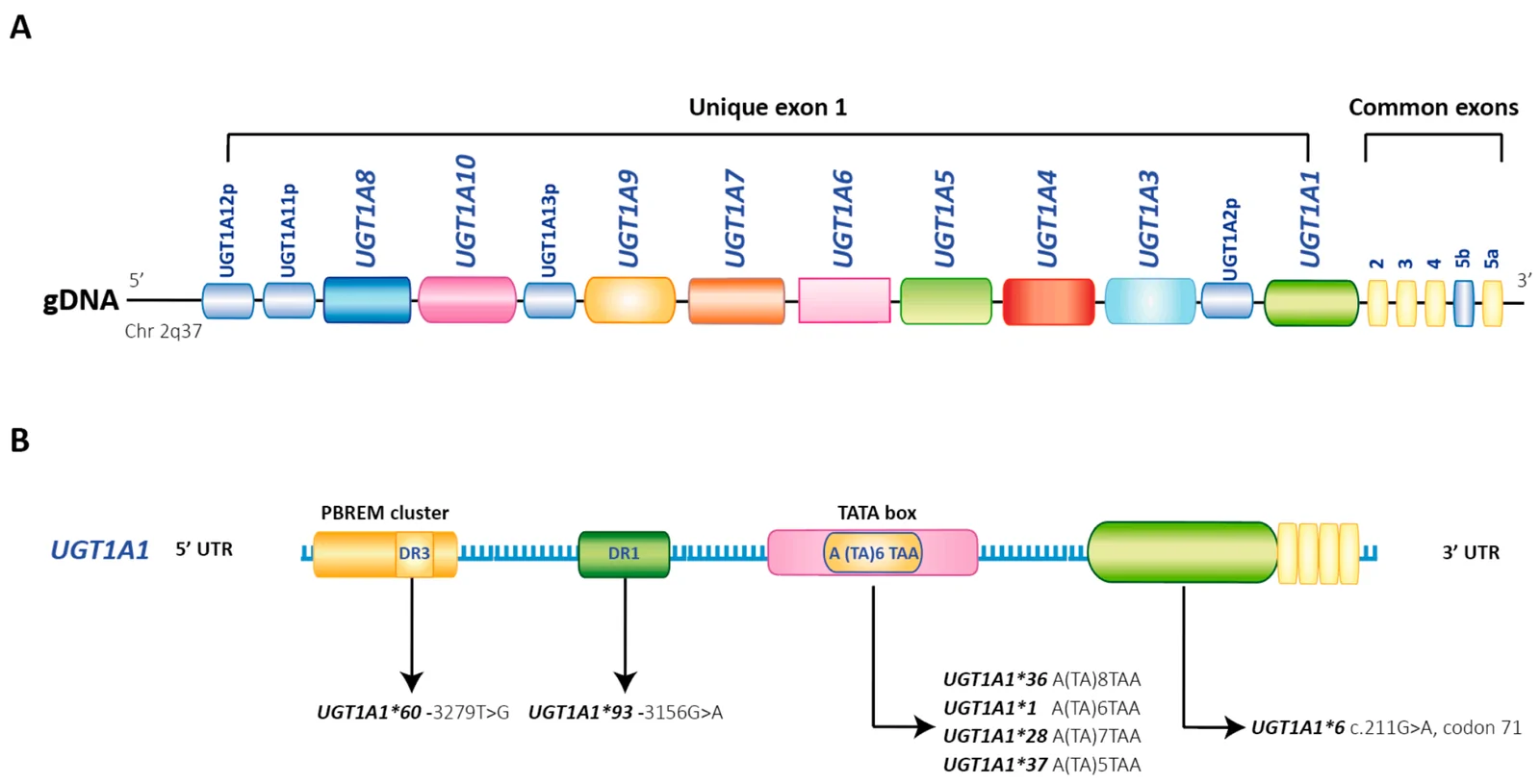
Schematic representation of the human *UGT1A* gene locus. (**A**) The *UGT1A* gene locus (rs8175347) is located in chromosome 2q37 and consists of multiple alternative unique coding exon sequences at the 5’ end (which collectively constitute exon 1) and the common exons 2-4 and 5a at the 3’ end. Each unique exon contains its own promoter site and it is joined with the common exon 2-5a sequence through a mechanism of splicing to produce different *UGT1A* isoforms. (**B**) The panel shows the TATA-box in the *UGT1A* promoter. Variations in the number of thymine-adenine (TA) repeats vary from 5 (TA)_5_TAA to 8 (TA)_8_TAA, with 6 repeats representing the wild-type allele associated with normal enzyme activity. The point mutation UGT1A1*6 (c. 211G > A, G71R) is depicted in the *UGT1A1* gene locus. Locations of two SNPs are shown in the *UGT1A1* upstream promoter (UGT1A1*60 c.-3279T > G and UGT1A1*93 c.-3156G > A). Alterations in [TA] repeats as well as switches of amino acids result in decreased/increased UGT1A1 activity.

The *UGT1A* locus consists of multiple alternative unique coding exon sequences at the 5’ end, which together constitute exon 1, and the common exons 2–4 and 5a at the 3’ end. Each exon has its own promoter site and allows the transcription of nine unique UGT1A enzymes. Protein isoforms originate by exon sharing, which results in enzymes with an identical 246-amino acid C-terminal region and unique N-terminal ends that provide functional diversity (Figure 1). UGT1A-spliced isoforms (named isoforms 2 or UGT1As_i2), although enzymatically inactive, may be generated when exon 5 (5b) is used rather than or in addition to exon 5a [11]. Moreover, four *UGT1A* pseudogenes have been identified, known as UGT1A2p, 11p, 12p and 13p [12,13].

The UGT1A enzyme is primarily expressed in the liver and in the gastrointestinal tract and is responsible for the conjugation and metabolism of bilirubin and other lipophilic substrates, such as steroids, toxins, and drugs [12,14].

UGT1A1 is a transmembrane protein, present in the endoplasmic reticulum, with two binding sites, one for substrate and another one for uridine diphosphate glucuronic acid (UDP-GlcA). The glucuronidation reaction occurs by second-order nucleophilic substitutions. First, the molecule of glucuronic acid (GlcA) is transferred to one of the carboxyl groups on the substrate. Then, another GlcA is added to the second carboxyl group and leads to the formation of diglucuronide metabolite and UDP. Glucuronides are hydrophilic compounds and can be readily excreted by active transport systems and eliminated in the bile and urine [15].

### UGT1A1 Variants

The *UGT1A1* gene is highly polymorphic and, so far, over 135 genetic variants have been reported. Polymorphisms in the *UGT1A1* gene lead to decreased enzyme activity, translating into interpatient differences in the pharmacokinetics of UGT1A1 substrates, including anticancer drugs [16].

Table 1 provides an overview of *UGT1A1* variants and their impact on metabolic function. Two regions of the *UGT1A1* gene, the promoter and exon 1, are involved in its polymorphisms. The promoter region contains a variable number of thymine–adenine [TA] dinucleotide repeats, known as a TATA box, where the polymorphisms arise depending on the number of [TA] repeats. The most common polymorphism is UGT1A1*28, characterized by seven TA repeats (TA_7_) instead of six (TA_6_), which presents in the reference allele UGT1A1*1. This change, described as a transition from A(TA)_6_TAA to A(TA)_7_TAA in the 5′ promoter region, leads to an approximately 70% reduction in the transcriptional activity of the *UGT1A1* gene promoter [17,18].

This variant occurs at a frequency of 37–56% in Africans, 37–40% in Latino populations, and 26–32% in Caucasians, while it is less common in Asian populations (0.09–0.4) [8].

Other *UGT1A1* variants involving the TATA box include UGT1A1*36 and UGT1A1*37. The UGT1A1*36 variant, characterized by a transition from A(TA)_6_TAA to A(TA)_5_TAA, results in increased promoter activity and has been associated with a reduced risk of neonatal hyperbilirubinemia [19]. In contrast, the UGT1A1*37 allele involves an A(TA)_6_TAA to A(TA)_8_TAA transition and impairs promoter activity. Both UGT1A1*36 and UGT1A1*37 are commonly found in African populations, with estimated allele frequencies of 0.03–0.10 and 0.02–0.07, respectively [20].

The allele variants generated by polymorphism in exon 1 are UGT1A1*6, UGT1A1*60 and UGT1A1*93. UGT1A1*6 causes a substitution of a Glycine with an Arginine in codon 71 of exon 1 (p.G71R, c. 211G > A), and it is commonly observed among Asian populations with an allele frequency of around 15–30% [21]. Patients homozygous for this variant have 32% of normal enzymatic activity and can be responsible for non-physiologic neonatal hyperbilirubinemia [22]. The variant UGT1A1*60 (-3279T > G, rs4124874) is a polymorphic mutation located in the phenobarbital-responsive enhancer module (gtPBREM). This variant is frequently observed in neonates with unconjugated neonatal hyperbilirubinemia. Nearly 60% of transcriptional activity is reduced in mutant gtPBREM with UGT1A1*60 [23]. Moreover, a study by Sugatani et al. showed that UGT1A1*60 may contribute to the development of mild hyperbilirubinemia in individuals with Gilbert’s syndrome [24].

The UGT1A1*93 (-3156G > A, rs10929302) variant is located in exon 1 between the TATAA box and gtPBREM, and has been described in strong linkage disequilibrium with UGT1A1*28 [25].

According to the *UGT1A1* genotype, individuals heterozygous for one decreased-function allele (e.g., UGT1A1 *1/*28) are predicted to be intermediate metabolizers (IM), while homozygous subjects (e.g., UGT1A1 *28/*28) are predicted to be poor metabolizers (PM) [26] (Table 2). Therefore, reduced UGT1A1 metabolic activity may lead to an increased risk of drug toxicity induced for drugs undergoing UGT1A1-mediated glucuronidation as the major elimination pathway.

Additionally, *UGT1A1* genetic variants may affect bilirubin elimination and cause hyperbilirubinemia, as in Crigler–Najjar syndrome and Gilbert’s syndrome [17]. These disorders involve aberrations in bilirubin conjugation due to a deficiency in bilirubin uridine diphosphate glucuronosyltransferase. Crigler–Najjar syndrome is a rare severe autosomal recessive inherited disease that leads to congenital non-hemolytic jaundice. This syndrome is caused by rare mutations in the *UGT1A1* gene, generating a complete or partially inactivated enzyme. Inactivation of the enzyme leads to severe hyperbilirubinemia that can cause irreversible brain damage, as encephalopathy (kernicterus) [5].

On the other hand, Gilbert’s syndrome is a common inherited disorder with an incidence of about 3–8.6% of the population and is characterized by mild hyperbilirubinemia (serum bilirubin levels range: 1–5 mg/dL). Patients with Gilbert’s syndrome are commonly homozygous carriers of the UGT1A1*28 polymorphism, which results in lower *UGT1A1* gene expression and decreased activity of the bilirubin uridine diphosphate glucuronosyltransferase enzyme [17].

## 3. UGT1A1 Substrates and Inhibitors in Anti-Cancer Therapy

**Figure 2:**
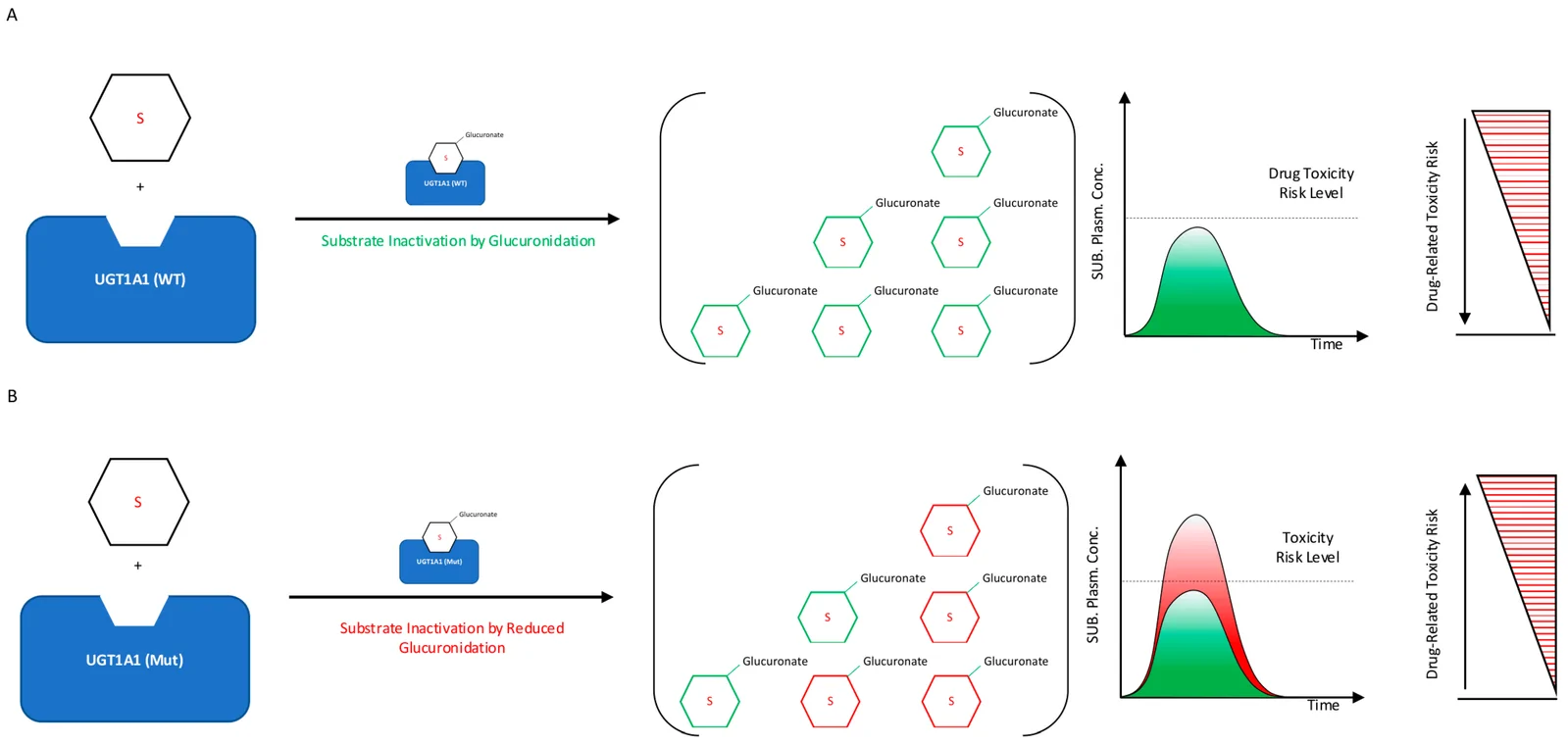
*UGT1A1* polymorphisms and cancer drug toxicities. (**A**) Substrates (S) are inactivated by *UGT1A1* wild-type (WT) enzyme through a Glucuronidation mechanism (green hexagon) that makes them less toxic and more water-soluble for excretion; (**B**) *UGT1A1* polymorphisms (Mut) decreased UGT1A1 enzyme activity, resulting in a reduced inactivation of the substrate and its consequent increasing plasma concentration, which leads to a major risk of cancer drug toxicities.

Several anticancer drugs are substrates or inhibitors of the UGT1A1 enzyme. For drugs that undergo UGT1A1-mediated glucuronidation as the main elimination pathway, specific variations in the *UGT1A1* sequence reduce enzymatic metabolic capacity, leading to high drug concentrations and eventually to an increased risk of drug-induced toxicity (Figure 2).

**Figure 3:**
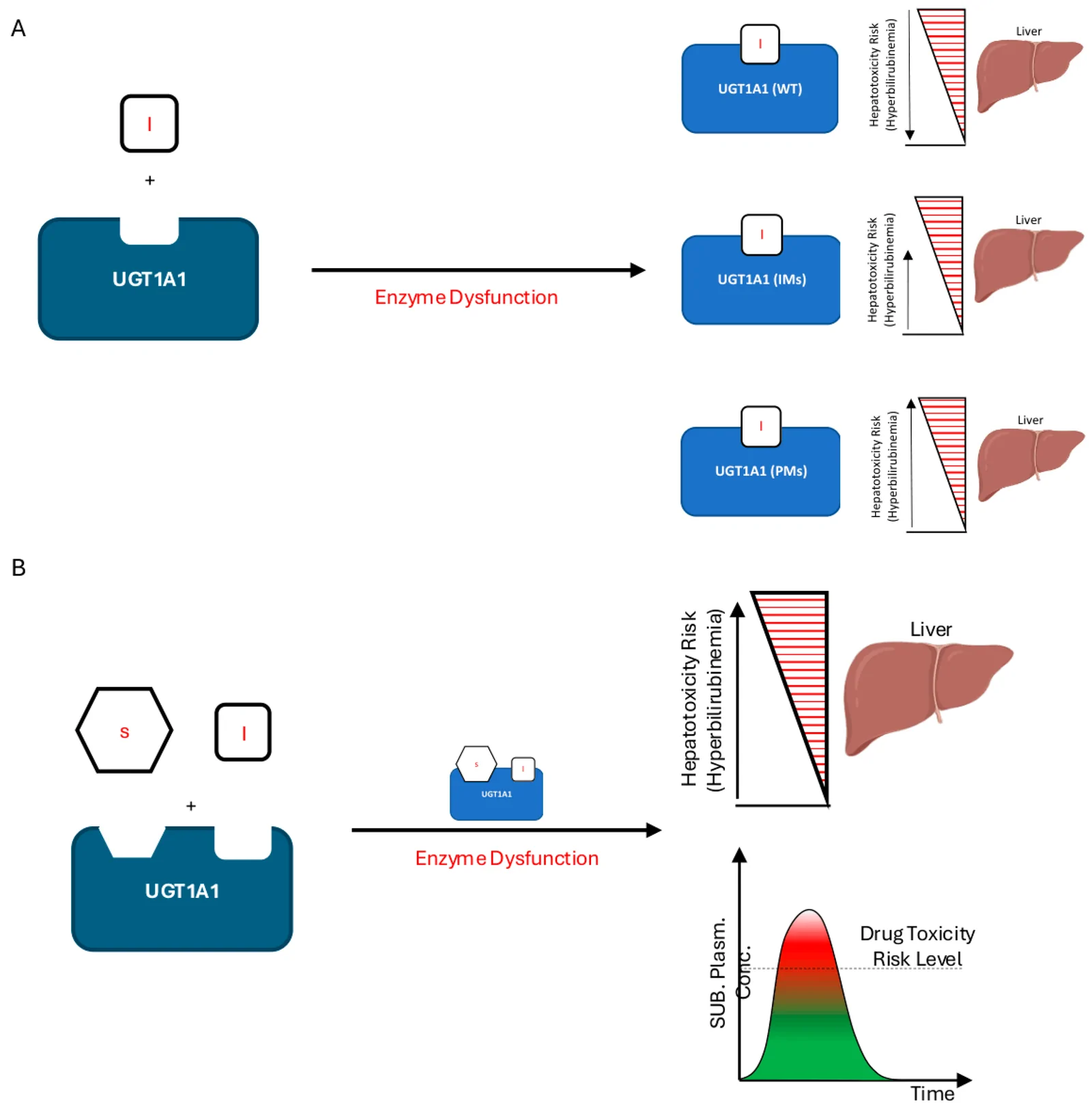
Association of UGT1A1 metabolic activity with liver and drug toxicity risk. (**A**) UGT1A1 inhibitors (I) lead to a hepatoxicity level dependent on UGT1A1 enzyme metabolic activity, which varies among wild-type (WT), intermediate (IMs), and poor (PMs) metabolizers. (**B**) Combined exposure to UGT1A1 substrates (S) and inhibitors (I), along with impaired UGT1A1 enzyme function, increases susceptibility to hepatotoxicity and adverse drug reactions.

On the other hand, molecules acting as UGT1A1 inhibitors can clinically mimic the homozygous phenotype for the alleles UGT1A1*28 and UGT1A1*6. Indeed, they can induce hyperbilirubinemia by competing with the enzyme’s binding site, and when used in combination with a UGT1A1 substrate can lead to hepatic toxicities [27] (Figure 3).

In both cases, genetic testing can be used to optimize genotype-guided dosing, thus preventing or mitigating potentially severe toxicities while maintaining treatment efficacy.

### 3.1. UGT1A1 Substrates

UGT1A1 substrates represent a heterogeneous group of anticancer agents with different levels of evidence supporting the clinical relevance of pharmacogenetic variability. While the association between reduced-function *UGT1A1* variants and irinotecan-related toxicity is well established, evidence for other UGT1A1 substrates remains limited, heterogeneous, or mainly based on pharmacokinetic studies. Differences in study design, treatment schedules, patient populations, and toxicity endpoints contribute to variability across studies and explain the lack of uniform clinical recommendations. Consequently, the strength of pharmacogenetic evidence differs considerably among UGT1A1 substrates, with irinotecan currently representing the best-validated model for genotype-guided clinical implementation. In the following sections, we provide an overview of the pharmacokinetics, pharmacodynamics, and pharmacogenetics of UGT1A1 substrates currently used in anticancer therapy (Table 3).

Irinotecan is a topoisomerase I inhibitor, approved for the treatment of multiple cancers, including colorectal, pancreatic, and gastric tumors. Late-onset diarrhea, myelosuppression and asthenia are the three most relevant and dose-limiting adverse events that may require treatment interruption or dose reduction in patients treated with irinotecan [28]. By inhibiting topoisomerase I, it has a time-dependent/phase-specific cytotoxic effect that induces cell cycle arrest in the S-G2 phase [29,30].

Two to five percent of the administered irinotecan is activated to 7-ethyl-10-hydroxycamptothecin (SN-38) in liver and plasma [30]. SN-38 is a molecule 100- to 1000-fold more cytotoxic than irinotecan with an increased half-life of about 47-fold, and it is responsible for the biological effects of irinotecan [28]. In the liver, about 70% of the active metabolite is converted to inactive glucuronide SN-38 (SN-38G) by uridine diphosphate glucuronosyltransferases (UGTs). Glucuronidation increases solubility and facilitates SN-38 excretion into the bile and then the urine [29].

Several UGT subtypes are involved in detoxification, but a major role is suggested for UGT1A1 [31]. UGT1A1 poor metabolizers (PMs), are at higher risk of toxicity when treated with irinotecan and they may display mild hyperbilirubinemia [32]. Genetic variants UGT1A1*6 and UGT1A1*28 are associated with increased SN-38 levels and with a greater risk of drug-induced toxicities [33]. Although increased SN-38 exposure clearly correlates with toxicity risk, the impact of the UGT1A1 genotype on irinotecan efficacy remains less defined. Some studies have suggested that higher SN-38 exposure may enhance antitumor activity, whereas others failed to demonstrate a consistent association between UGT1A1 variants and treatment response or survival outcomes. Therefore, current evidence primarily supports UGT1A1 genotyping as a toxicity-risk stratification tool rather than an efficacy-guiding biomarker.

Liposomal irinotecan (nal-IRI) is a formulation of irinotecan encapsulated in a lipid bilayer vesicle that improves bioavailability. It is approved for the treatment of patients with advanced pancreatic cancer [34]. Nal-IRI has a higher exposure and longer plasma half-life compared to its non-liposomal counterpart, resulting in a different clinical efficacy and safety profile [35].

Sacituzumab govitecan (SG) is an antibody–drug conjugate (ADC) approved for the treatment of advanced triple-negative and hormone-receptor-positive (HR+) breast cancer [36,37]. Sacituzumab govitecan was constructed by site-specific conjugation of SN-38 with a humanized monoclonal antibody (hRS7) directed against trophoblastic cell surface antigen 2 (Trop-2) [38]. Seven to eight molecules of SN-38 are covalently bound to hRS7 via a pH-dependent hydrolyzable CL2A linker [39]. When SG interacts with surface receptor Trop-2, the cytotoxic payload is internalized into tumor cells, inducing DNA double-strand breaks and apoptosis during cell cycle S-phase [40,41]. Free SN-38 can also exert its effect on neighboring tumor cells through the so-called bystander effect [38].

The most common adverse reactions in patients treated with SG are neutropenia, representing the dose-limiting toxicity, diarrhea, nausea, fatigue, and anemia [16,42].

The mean distribution volume was 0.045 L/kg after a single dose of 10 mg/kg [43]. Average half-lives of SG and free SN-38 were 16 and 18 h, respectively. SN-38 levels were largely eliminated at a rate of ~50% per day within 3 days of treatment. Drug concentrations typically increase proportionally with increasing dose [44]. No metabolism studies have been conducted; however, SN-38 is metabolized via UGT1A1 mainly in the liver [44]. Given that sacituzumab govitecan delivers an SN-38 payload, UGT1A1 variability represents a biologically plausible determinant of drug exposure and toxicity. However, the pharmacogenetic evidence supporting sacituzumab govitecan is considerably less mature than that available for conventional irinotecan. Current data are derived predominantly from post hoc analyses of registration trials and retrospective observational studies rather than prospective pharmacogenetic investigations specifically designed to evaluate genotype–toxicity associations. Although these studies consistently suggest a higher incidence of hematological and gastrointestinal toxicities among carriers of reduced-function *UGT1A1* variants, particularly UGT1A1*28 homozygotes, the available evidence remains insufficient to support genotype-guided dose adjustment in routine clinical practice. Furthermore, the pharmacokinetic profile of antibody–drug-conjugate-mediated SN-38 delivery differs substantially from that of conventional irinotecan, making direct extrapolation of irinotecan-based dosing recommendations inappropriate. Prospective studies are therefore needed to clarify the clinical utility of *UGT1A1*-guided treatment strategies in patients receiving sacituzumab govitecan.

Etoposide (VP-16) is a topoisomerase II inhibitor used to treat a variety of solid tumors and hematological malignancies. This drug inhibits its target by increasing the steady-state concentration of topoisomerase II covalent complexes. This converts topoisomerases into physiological toxins, introducing high levels of transient protein-associated breaks and eventually leading to cell death by apoptosis [45]. The main adverse effects of this agent include myelosuppression, which is dose-related and dose-limiting, nausea and vomiting.

When administered intravenously the drug disposition is biphasic, with a distribution half-life of approximately 1.5 h and a terminal elimination half-life of 4 to 11 h. The clearance is partly related to creatinine clearance. Part of the administered drug is excreted in the urine, a small amount in bile and a large proportion in the liver. Hepatic metabolization is performed by CYP3A4 and the UGT1A1. Etoposide glucuronide accounts for 15–35% of the administered dose [46].

Axitinib is a selective second-generation inhibitor of vascular endothelial growth factor (VEGFR) isoforms 1, 2, and 3, approved for the treatment of renal cell carcinoma. It binds the intracellular tyrosine kinase domains of VEGFR, blocking downstream signaling. In preclinical studies, axitinib significantly inhibited VEGF-mediated endothelial cell adhesion and migration, cellular phosphorylation of VEGFR-2, tube formation and vascular permeability, proving to be more selective than other multitargeted tyrosine kinase inhibitors [47]. At low nanomolar concentrations, axitinib is also an inhibitor of platelet-derived growth factor receptors (PDGFR-β) and tyrosine-protein kinase KIT, CD117 (c-Kit) [47].

The drug is orally administered. It is transported in plasma bound to albumin and has a short half-life, between 2.5 and 6.1 h [48]. The metabolism of axitinib is primarily mediated by CYP3A4/5 and to a lesser extent by CYP1A2, CYP2C19, and UGT1A1. These enzymes are responsible for generating other oxidative products and glucuronides such as M12, the sulfoxide metabolite, and M7, the glucuronide conjugate [49]. Axitinib is eliminated via the hepatobiliary system, with minimal renal excretion. As axitinib metabolism is also mediated by UGT1A1, genetic variations in this gene may contribute to pharmacogenetic variability and they may interfere with efficacy and toxicities. However, in all pharmacological clinical trials, none of the polymorphisms analyzed were significant in explaining the pharmacokinetic variability of axitinib [48].

Belinostat is a histone deacetylase inhibitor (HDAC) approved by the FDA for the treatment of relapsed or refractory peripheral T-cell lymphoma (PTCL) [50]. HDAC inhibition results in an accumulation of acetylated histones and a more relaxed chromatin structure, which promotes transcription of genes involved in arrest of cell growth, differentiation and apoptosis of cancer cells [51]. Nausea, vomiting, lethargy, fatigue, constipation, flushing, and diarrhea are the most common adverse effects [50]. After intravenous administration, belinostat is rapidly eliminated, with a half-life ranging from 0.3 to 3.5 h [52]. This drug is primarily eliminated through hepatic metabolism via UGT1A1-mediated glucuronidation and to a lesser extent by CYP2A6, CYP2C9, and CYP3A4 enzymes to form belinostat amide and belinostat acid. Less than 2% of the drug is excreted unchanged in urine. Poor UGT1A1 metabolizer may be exposed to higher plasma concentrations of the drug, possibly increasing the incidence of severe toxicity. Phenotypic consequences of *UGT1A1* variants have been demonstrated in preclinical and clinical studies, and the most commonly reported *UGT1A1* genetic variants associated with altered enzyme expression or activity are UGT1A1*6, UGT1A1*28, UGT1A1*60, and UGT1A1*93 [53]. Specifically, UGT1A1*28 and UGT1A1*60 variant alleles were significantly associated with higher belinostat exposure and an increased risk of haematotoxicity, such as thrombocytopenia and neutropenia [54].

### 3.2. Functional UGT1A1 Anti-Cancer Inhibitor

UGT1A1 inhibition may lead to clinically significant drug–drug interaction (DDI) due to its key role in the detoxification of several drugs with narrow therapeutic windows. Particularly, UGT1A1 inhibitors may have a greater impact in UGT1A1 PM, as these individuals already have reduced basal enzymatic activity (Figure 3B). Currently, there is no FDA-approved label for any of the UGT1A1 inhibitors with specific dosing recommendations, but precautions are suggested for PM UGT1A1 patients [55].

Among anti-cancer drugs, many small molecules are potent inhibitors of UGT1A1 (Table 4) [27,56]. Several in vitro and in vivo studies showed that tyrosine kinase inhibitors (TKIs) can alter the hepatic elimination of co-administered drugs through competitive, non-competitive or mixed inhibition, causing a wide range of undesirable side effects. Based on K_i_ inhibition-constant values and their plasma concentrations, TKIs exhibit strong to moderate inhibitory effects on UGT1A1 [56].

In this context, lapatinib, nilotinib, pazopanib, regorafenib and sorafenib are potent inhibitors of bilirubin glucuronidation, whereas imatinib acts as an intermediate inhibitor and erlotinib and gefitinib are weak inhibitors [6,8].

Lapatinib is indicated for the treatment of adult patients with breast cancer whose tumors overexpress Human Epidermal Receptor 2 (HER2) [57]. It is a potent competitive inhibitor of UGT1A1. A clinical study showed that co-administration of lapatinib with irinotecan resulted in an approximately 40% increase in the biodisponibility AUC of SN-38 [58].

Nilotinib (2G) and imatinib (1G) are TKIs approved for treatment of patients with BCR::ABL1-positive chronic myelogenous leukemia (CML). Imatinib is also indicated for the treatment of gastrointestinal stromal tumors (GIST). Co-administration of nilotinib and SN-38 may significantly increase the biodisponibility (AUC) of SN-38, in addition to the high rate of hyperbilirubinaemia observed in UGT1A1*28-homozygous CML patients [27]. It is known that nilotinib should be used with caution in patients with Gilbert’ syndrome [59]. An AUC increase of 3% has been observed when used as a competitive inhibitor in CML patients, while only a few cases of GIST patients are reported in the literature [56].

Pazopanib is a multiple TKI indicated for the treatment of patients with advanced renal cell carcinoma and advanced soft tissue sarcoma. It has anti-angiogenic activity through inhibition of vascular endothelial growth factor receptor (VEGFR), fibroblast growth factor receptor (FGFR), and platelet-derived growth factor receptor (PDGFR) [60]. A study of 116 individuals showed that UGT1A1*28/*28 PMs were significantly more likely to develop hyperbilirubinemia compared to other genotypes [61].

Regorafenib is a multikinase inhibitor approved for treating advanced colorectal cancer. Kinetic experiments confirmed that regorafenib is a potent inhibitor of human liver microsomal β-estradiol glucuronidation, an established surrogate for bilirubin glucuronidation [6].

Sorafenib inhibits tumor cell proliferation through the Raf/extracellular signal-regulated kinase (ERK) kinase (MEK)/ERK signaling pathway, and tumor angiogenesis by targeting VEGFRs 2 and 3 and PDGFR. It is indicated for the treatment of hepatocellular carcinoma, renal cell carcinoma and thyroid carcinoma [62]. A single-centre dose-escalation Phase I study showed that sorafenib 400 mg twice daily increases exposure to SN-38 [63].

Gefitinib and erlotinib are indicated for the treatment of patients with advanced non-small-cell lung cancer with activating EGFR mutations. Both drugs are weak inhibitors, and they are unlikely to cause clinically significant DDI through inhibition of glucuronidation [64]. Recently, a possible association with hyperbilirubinaemia has also been shown for sunitinib, a TKI used for the treatment of adult patients with GIST, advanced RCC and pancreatic neuroendocrine tumors (pNET). However, its relation remains unexplored [65].

Lastly, everolimus, a mammalian target of rapamycin (mTOR) inhibitor used in HR+ advanced breast cancer, p-NET, neuroendocrine tumors and RCC, has been characterized as an UGT1A1 inhibitor. The calculated Ki value was very low, indicating that everolimus is unlikely to cause clinically significant DDI by inhibiting UGT1A1 [66].

## 4. Clinical Impact of *UGT1A1* Polymorphisms: From Pharmacokinetics to Clinical Practice

Although UGT1A1 testing is not yet mandatory in routine clinical practice, understanding the relationship between *UGT1A1* genotypes and chemotherapy-induced toxicities can help optimize patient care. Current evidence supports the clinical use of UGT1A1 genotyping mainly as a tool for toxicity risk stratification, particularly before irinotecan-based treatments, whereas its role in guiding efficacy-based decisions remains uncertain. In clinical practice, testing may be considered before treatment initiation in patients receiving high-dose irinotecan or in those with additional risk factors for severe toxicity, including older age, female sex, poor performance status, or concomitant therapies affecting UGT1A1 activity.

A substantial body of evidence exists regarding the correlation between irinotecan, UGT1A1 polymorphisms and the onset of hematological and non-hematological toxicities.

In the CAIRO study, UGT1A1 *28/*28 homozygous patients receiving capecitabine and irinotecan experienced higher rates of febrile neutropenia (18.2%) compared to wild-type (*1/*1, 1.5%) and heterozygous (*1/*28, 6.5%) patients. Grade 3–4 neutropenia was also more frequent in the homozygous group (66.7% vs. 15.2% and 22.6%) [67]. Similar findings were reported in the PETACC-3 and MECC trials, with *28/*28 patients showing earlier onset and higher incidence of neutropenia, particularly at higher irinotecan doses (>150 mg/m^2^) and in female patients [68,69]. Early-onset dose-dependent neutropenia in homozygous patients was also confirmed in a subsequent meta-analysis, with no significant effects observed in patients treated with low doses of irinotecan (<150 mg/mq) [69,70]. The association between *UGT1A1* polymorphisms and severe diarrhea remains unclear. While Yu et al. found a significant association between UGT1A1*28 genotypes and grade 3/4 diarrhea in a prospective multicenter trial, the PETACC-3 trial showed a weak association, and the MECC trial did not support this correlation [68,69,71]. These discrepancies highlight that genotype alone is unlikely to fully predict toxicity, as treatment schedule, dose intensity, ethnicity, comorbidities, and concomitant medications also contribute to individual variability.

Regarding clinical outcomes, data are mixed: the CAIRO trial found no genotype-related differences in tumor response, while the MECC trial and Yu et al. suggested shorter progression-free disease and lower overall survival rates in *28/*28 patients, likely due to dose reductions or treatment interruptions [67,69,71]. Therefore, the *UGT1A1* genotype should currently be considered primarily a safety biomarker rather than a validated predictor of irinotecan efficacy.

The PURE FIST trial suggests that genotyping could guide dose adjustments: escalating doses in wild-type patients while reducing doses for heterozygous and homozygous patients may prevent discontinuation and improve outcomes [72,73]. However, prospective validation of genotype-guided dosing algorithms in different tumor types and treatment settings is still required before universal implementation.

Building on this evidence, similar genotype–toxicity associations have been described for liposomal irinotecan (nal-IRI), a formulation used in combination with 5FU/LV in pretreated metastatic pancreatic cancer. Nal-IRI retains the typical toxicity profile of irinotecan (notably neutropenia and diarrhea), but with some differences in incidence and timing. Analyses from the NAPOLI-1 trial and subsequent studies have demonstrated that carriers of reduced-function *UGT1A1* alleles (*28/*28, and *6 in Asian populations) are at increased risk of high-grade neutropenia and dose-dependent hematological events when treated with nal-IRI [74,75]. According to the FDA-approved label, UGT1A1 *28/28* patients should receive nal-IRI at a recommended initial dose of 50 mg/m^2^, with the option to escalate to 70 mg/m^2^ in subsequent cycles if tolerated [76]. This represents one of the clearest examples of genotype-informed dosing currently available in oncology practice.

UGT1A1 polymorphisms also influence SG toxicity. In the phase 3 ASCENT trial, *28/*28 patients had higher rates of grade 3 neutropenia (59%) compared to *1/*28 (47%) and wild-type (53%). Febrile neutropenia, anemia, and diarrhea were more frequent in the homozygous group, which also had higher treatment discontinuation rates [77]. Similar trends were observed in TROPICS-02, IMMU-132-01, and TROPHY trials, with *28/*28 patients showing the highest incidence of grade 3–4 hematologic toxicities and more frequent dose reductions or discontinuations [37,78]. Despite these findings, UGT1A1-guided dose adjustment for SG is not currently standardized, and further prospective analyses are needed to define its clinical utility.

*UGT1A1* polymorphisms may also increase etoposide-related toxicity. Homozygous carriers of *6 or *28 alleles experienced higher rates of nephrotoxicity, prolonged febrile neutropenia, and gastrointestinal toxicities, including nausea, anorexia, and hepatobiliary effects [79,80]. According to other evidence, neutropenia and thrombocytopenia risk is also dose-dependent in patients carrying one or two *28 alleles [54].

With regards to UGT1A1 inhibitors, gene polymorphisms may influence axitinib serum levels, potentially raising the risk of adverse events such as hypertension, proteinuria and anorexia, as shown in a study by Narita et al. on metastatic renal cancer patients [81].

Several tyrosine kinase inhibitors (TKIs) also interact with UGT1A1 pathways. Lapatinib has been identified as an inhibitor of UGT1A1-mediated glucuronidation, a mechanism that contributes to hepatotoxicity and hyperbilirubinemia [82]. Nilotinib is strongly associated with bilirubin elevations, particularly in carriers of reduced-function alleles such as *28 [83]. Pazopanib is a potent inhibitor of UGT1A1, and isolated benign hyperbilirubinemia is frequently reported in patients with Gilbert’s syndrome [65]. Regorafenib and sorafenib inhibit UGT1A1 in vitro and have been linked to hyperbilirubinemia and drug–drug interactions, as well as hypertension, fatigue, and hand–foot skin reactions [65,84].

Overall, implementation of *UGT1A1* testing should currently be individualized. Routine testing is best supported before irinotecan and nal-IRI administration, while evidence for other substrates and ADCs remains insufficient to recommend universal screening. Integration of genotype results with clinical variables, treatment dose, ethnicity, and concomitant medications will likely be necessary to maximize the clinical utility of UGT1A1-guided therapy.

## 5. Guidelines for UGT1A1 Testing in Clinical Practice

Imatinib has been shown to interfere with glucuronidation pathways, potentially affecting UGT substrates and causing toxicities such as edema, gastrointestinal intolerance, muscle cramps, and liver enzyme elevations [85]. Similarly, erlotinib and gefitinib have been listed among agents with potential UGT1A1 interactions, although clinical evidence of a clear genotype–toxicity relationship is limited; their typical adverse events include rash, diarrhea, and hepatic enzyme abnormalities [86,87].

The Italian Association of Medical Oncology–Italian Pharmacology Society (AIOM-SIF), Clinical Pharmacogenetics Implementation Consortium (CPIC), Dutch Pharmacogenetics Working Group (DPWG), European Medicines Agency (EMA), U.S. Food and Drug Administration (FDA), National Comprehensive Cancer Network (NCCN), and French National Network of Pharmacogenetics (RNPGx) provide valuable guidelines on the appropriate use and interpretation of pharmacogenomic testing in oncology. Specifically, these organizations have been compared to determine whether recommendations exist for UGT1A1 substrates and UGT1A1 inhibitors in anticancer therapy (Table 5).

The AIOM-SIF strongly recommends a 30% dose reduction of irinotecan in UGT1A1*28 homozygotes, but provides no guidance for other UGT1A1-metabolized drugs [88].

The CPIC does not currently provide genotype-adjusted dosing guidelines but classifies irinotecan as a ‘level A’ drug, indicating sufficient evidence to use genetic profiles for individualized therapy, and belinostat as a ‘level B’ drug, for which genetic information could potentially inform prescribing [89].

The DPWG provides recommendations only for irinotecan, advising a 30% starting dose reduction in UGT1A1*28 homozygotes, with subsequent dose escalation guided by neutrophil counts if tolerated. No dose adjustments are recommended for heterozygotes or other drugs [90].

The EMA similarly recommends an initial dose reduction of irinotecan in *poor metabolizers, with no further guidance for other UGT1A1 substrates [91].

FDA labels for irinotecan, belinostat, pazopanib, and nilotinib include pharmacogenetic warnings. For irinotecan, UGT1A1*28 homozygosity predicts severe neutropenia [92]. Belinostat labels recommend a reduced starting dose of 750 mg/m^2^ in *28/*28 patients [93]. Moreover, pazopanib and nilotinib indicate a higher risk of hyperbilirubinemia in poor metabolizers [94].

NCCN provides no formal dosing guidance but advises caution when administering irinotecan to patients with Gilbert’s syndrome [95].

RNPGx proposes a decision tree stratifying patients as poor (PMs), intermediate (IMs), or normal metabolizers (NMs), with an initial dose reduction recommended only when prescribing irinotecan [96].

Despite broad agreement regarding the clinical relevance of UGT1A1 polymorphisms for irinotecan-associated toxicity, important differences remain among international pharmacogenetic guidelines. These discrepancies primarily reflect differences in the interpretation and quality of the available evidence, the irinotecan dose thresholds considered clinically relevant, the balance between toxicity prevention and maintenance of treatment efficacy, and the intended scope of each guideline. Consequently, recommendations regarding pre-treatment genotyping, patient selection, and genotype-guided dose adjustment remain only partially harmonized across scientific societies.

## 6. Conclusions

In this review, we have explored the physiological and pharmacological role of the UGT1A1 enzyme, focusing on how its genetic polymorphisms impact pharmacokinetic parameters and the clinical outcomes of antitumoral drugs. We have analyzed several anticancer agents that are either substrates or inhibitors of UGT1A1 and discussed how genetic variability may influence drug metabolism, treatment exposure, and susceptibility to toxicity. Indeed, *UGT1A1* polymorphisms show important clinical implications in oncology with a pharmacokinetic impact associated with toxicity profiles of various anticancer therapies. Extensive evidence, especially derived from irinotecan-based treatments, demonstrated that genetic variations in the *UGT1A1* gene can markedly affect drug metabolism, leading to individual differences in both efficacy and risk of severe adverse events. Indeed, identifying patients with reduced-function alleles (e.g., *28/*28) can help to guide dose-adjustments, preventing severe hematological or gastrointestinal toxicities and improving individualized treatment planning. Despite growing data supporting this dose-adjustment effectiveness according to *UGT1A1* genotype, routine implementation of polymorphisms identification in clinical practice remains inconsistent due to the differences among international guidelines and limited prospective validation.

Overall, the clinical relevance of UGT1A1 variability differs substantially among anticancer agents. For irinotecan, evidence supporting toxicity prediction is robust, although optimal implementation strategies remain debated. Conversely, for newer SN-38-based antibody–drug conjugates, such as sacituzumab govitecan, and other UGT1A1 substrates, available data remain insufficient to define validated genotype-guided treatment approaches. Differences in study design, patient populations, treatment schedules, and clinical endpoints contribute to uncertainty and explain the variability among current recommendations.

To date, testing is most robustly supported for irinotecan, whereas guidance for other UGT1A1 substrates and inhibitors is still emerging. Further prospective studies are needed to establish evidence-based dose adjustments and to integrate comprehensive pharmacogenetic strategies across a broader range of anticancer agents, with the ultimate goal of improving patient outcomes and quality of life.

## Figures and Tables

**Table 1:** *UGT1A1* allele variants and their predicted impact on enzymatic functioning.

*UGT1A1* Allele	dbSNP Reference	Variant Type	AA Change	Location	UGT1A1 Expression/Activity
UGT1A1*1	rs8175347	A(TA)6TAA	-	Promoter	Normal (100%)
UGT1A1*28	rs8175347	A(TA)7TAA	-	Promoter	Reduced expression
UGT1A1*36	rs8175347	A(TA)5TAA	-	Promoter	Increased expression
UGT1A1*37	rs8175347	A(TA)8TAA	-	Promoter	Reduced expression
UGT1A1*6	rs4148323	c.211G > A	p.(Gly71Arg)	Exon 1	Reduced activity
UGT1A1*60	rs4124874	c.-3279T > G	-	Exon 1	Reduced expression
UGT1A1*93	rs10929302	c.-3156G > A	-	Exon 1	Reduced expression

Table legend: *UGT1A1*—Uridine diphosphate glucuronosyltransferase 1A1; dbSNP—Database of Single Nucleotide Polymorphisms; AA—Amino acid.

**Table 2:** *UGT1A1* genotype and predicted phenotype.

Predicted UGT1A1 Phenotype	Genotype
Normal metabolizer	*1/*1, *1/*36, *36/*36
Intermediate metabolizer	*1/*28, *1/*6, *1/*37, *6/*36, *28/*36, *36/*37
Poor metabolizer	*6/*6, *6/*28, *28/*28, *6/*37, *28/*37, *37/*37

**Table 3:** *UGT1A1* anti-cancer substrate.

Drug	Selected Indications	Clinically Relevant UGT1A1 Variants	Main Toxicities	*UGT1A1* Variants and Toxicity
Irinotecan	mCRC, SCLC, NSCLC, GC, OC	UGT1A1*28; UGT1A1*6	Neutropenia, diarrhea	Yes
Etoposide	AML, HL, NHL, GC, OC	UGT1A1*28	Myelosuppression, nausea, vomiting	UKN
Sacituzumab govitecan	mTNBC, mUC	UGT1A1*28	Nausea, diarrhea, fatigue, neutropenia and anaemia	Yes
Belinostat	PTCL	UGT1A1*28; UGT1A1*60	Nausea, vomiting, thrombocytopenia, neutropenia	Yes
Axitinib	aRCC	UKN	Diarrhea, hypertension, fatigue and nausea	UKN

Table legend: mCRC—metastatic colorectal cancer, SCLC—small-cell lung cancer; NSCLC—non-small-cell lung cancer; GC—gastric cancer; OC—ovarian cancer; AMLacute myeloid leukemia; HL—Hodgkin lymphoma; NHL—non-Hodgkin lymphoma; mTNBC—metastatic triple-negative breast cancer; mUCmetastatic urothelial cancer; PTCL—peripheral T-cell lymphoma; aRCC—advanced renal cell carcinoma; UKN—unknown.

**Table 4:** UGT1A1 anti-cancer inhibitors.

Drug	Target	Indications	UGT1A1 Inhibition	Induced Hyperbilirubinaemia
Lapatinib	HER2	HER2+ BC	Strong	Yes
Nilotinib	BCR::ABL1	CML	Strong	Yes
Pazopanib	Multiple kinases	aRCC and sarcoma	Strong	Yes
Regorafenib	Multiple kinases	mCRC	Strong	Yes
Sorafenib	Multiple kinases	HC, aRCC, mTC	Strong	UKN
Imatinib	BCR::ABL1; cKIT	CML, GIST	Intermediate	UKN
Erlotinib	EGFR	mNSCLC, mPC	Weak	Yes
Gefitinib	EGFR	aNSCLC	Weak	Yes
Sunitinib	Multiple kinases	GIST, aRCC, pNET	Weak	No
Everolimus	mTOR	aBC, pNET, RCC	Weak	No

Table Legend: HER2—Human Epidermal growth factor Receptor 2; BC—Breast Cancer; CML—Chronic Myeloid Leukemia; aRCC—Advanced Renal Cell carcinoma; mCRC—Metastatic Colorectal Cancer; HC—Hepatocellular Carcinoma; mTC—Metastatic Thyroid Cancer; GIST—Gastrointestinal Stromal Tumor; mNSCLC—Metastatic Non-Small-Cell Lung Cancer; mPC—Metastatic Pancreatic Cancer; aNSCLC—Advanced Non-Small-Cell Lung Cancer; pNET—Pancreatic Neuroendocrine Tumor; aBC—Advanced Breast Cancer; UKN—Unknown.

**Table 5:** Summary of recommendations about drug dosing according to UGT1A1 polymorphisms.

Organization	Drug	*UGT1A1* Variant	Recommendations
AIOM-SIF	Irinotecan	*28/*28	30% dose reduction; no guidance for other drugs
CPIC	Irinotecan	N/A	Level A: genetic profile can guide dosing
	Belinostat	N/A	Level B: evidence may inform prescribing
DPWG	Irinotecan	*28/*28	30% starting dose reduction; increase guided by neutrophil counts if tolerated; no adjustment for heterozygotes
EMA	Irinotecan	*28/*28	Initial dose reduction recommended; no guidance for other drugs
FDA	Irinotecan	*28/*28	Warning: predicts severe neutropenia
	Belinostat	*28/*28	Reduced starting dose: 750 mg/m^2^
	Pazopanib/Nilotinib	*28/*28	Increased risk of hyperbilirubinemia
NCCN	Irinotecan	Gilbert’s syndrome/*28	Exercise caution; no formal dose recommendation
RNPGx	Irinotecan	PM/IM/NM	Initial dose reduction only for PM; stratify patients by metabolism status

Table Legend: AIOM-SIF—Associazione Italiana di Oncologia Medica-Società Italiana di Farmacologia; CPIC—Clinical Pharmacogenetics Implementation Consortium; DPWG—Dutch Pharmacogenetics Working Group; EMA—European Medicines Agency; FDA: Food and Drug Administration; NCCN—National Comprehensive Cancer Network; RNPGx—Réseau National de Pharmacogénétique; N/A—not available; PM—poor metabolizer; IM—intermediate metabolizer; NM—normal metabolizer.

## Data Availability

No new data were created or analyzed in this study. Data sharing is not applicable to this article.

## References

[1] Letai A., Bhola P., Welm A.L. (2022). Functional precision oncology: Testing tumors with drugs to identify vulnerabilities and novel combinations. Cancer Cell.

[2] Di Lauro V., Barchiesi G., Martorana F., Zucchini G., Muratore M., Fontanella C., Arpino G., Del Mastro L., Giuliano M., Puglisi F. (2022). Health-related quality of life in breast cancer patients treated with CDK4/6 inhibitors: A systematic review. ESMO Open.

[3] Roden D.M., McLeod H.L., Relling M.V., Williams M.S., Mensah G.A., Peterson J.F., Van Driest S.L. (2019). Pharmacogenomics. Lancet.

[4] Cervantes A., Adam R., Rosello S., Arnold D., Normanno N., Taieb J., Seligmann J., De Baere T., Osterlund P., Yoshino T. (2023). Metastatic colorectal cancer: ESMO Clinical Practice Guideline for diagnosis, treatment and follow-up. Ann. Oncol..

[5] Kadakol A., Ghosh S.S., Sappal B.S., Sharma G., Chowdhury J.R., Chowdhury N.R. (2000). Genetic lesions of bilirubin uridine-diphosphoglucuronate glucuronosyltransferase (UGT1A1) causing Crigler-Najjar and Gilbert syndromes: Correlation of genotype to phenotype. Hum. Mutat..

[6] Miners J.O., Chau N., Rowland A., Burns K., McKinnon R.A., Mackenzie P.I., Tucker G.T., Knights K.M., Kichenadasse G. (2017). Inhibition of human UDP-glucuronosyltransferase enzymes by lapatinib, pazopanib, regorafenib and sorafenib: Implications for hyperbilirubinemia. Biochem. Pharmacol..

[7] Henriksen J.N., Bottger P., Hermansen C.K., Ladefoged S.A., Nissen P.H., Hamilton-Dutoit S., Fink T.L., Donskov F. (2020). Pazopanib-Induced Liver Toxicity in Patients With Metastatic Renal Cell Carcinoma: Effect of UGT1A1 Polymorphism on Pazopanib Dose Reduction, Safety, and Patient Outcomes. Clin. Genitourin. Cancer.

[8] Nelson R.S., Seligson N.D., Bottiglieri S., Carballido E., Cueto A.D., Imanirad I., Levine R., Parker A.S., Swain S.M., Tillman E.M. (2021). UGT1A1 Guided Cancer Therapy: Review of the Evidence and Considerations for Clinical Implementation. Cancers.

[9] Mackenzie P.I., Bock K.W., Burchell B., Guillemette C., Ikushiro S., Iyanagi T., Miners J.O., Owens I.S., Nebert D.W. (2005). Nomenclature update for the mammalian UDP glycosyltransferase (UGT) gene superfamily. Pharmacogenet. Genom..

[10] Meech R., Hu D.G., McKinnon R.A., Mubarokah S.N., Haines A.Z., Nair P.C., Rowland A., Mackenzie P.I. (2019). The UDP-Glycosyltransferase (UGT) Superfamily: New Members, New Functions, and Novel Paradigms. Physiol. Rev..

[11] Girard H., Levesque E., Bellemare J., Journault K., Caillier B., Guillemette C. (2007). Genetic diversity at the UGT1 locus is amplified by a novel 3′ alternative splicing mechanism leading to nine additional UGT1A proteins that act as regulators of glucuronidation activity. Pharmacogenet. Genom..

[12] Gong Q.H., Cho J.W., Huang T., Potter C., Gholami N., Basu N.K., Kubota S., Carvalho S., Pennington M.W., Owens I.S. (2001). Thirteen UDPglucuronosyltransferase genes are encoded at the human UGT1 gene complex locus. Pharmacogenetics.

[13] Innocenti F., Ratain M.J. (2004). “Irinogenetics” and UGT1A: From genotypes to haplotypes. Clin. Pharmacol. Ther..

[14] Court M.H., Zhang X., Ding X., Yee K.K., Hesse L.M., Finel M. (2012). Quantitative distribution of mRNAs encoding the 19 human UDP-glucuronosyltransferase enzymes in 26 adult and 3 fetal tissues. Xenobiotica.

[15] Memon N., Weinberger B.I., Hegyi T., Aleksunes L.M. (2016). Inherited disorders of bilirubin clearance. Pediatr. Res..

[16] Ibrahim R., Khoury R., Ibrahim T., Le Cesne A., Assi T. (2024). UGT1A1 Testing in Breast Cancer: Should it become routine practice in patients treated with antibody-drug conjugates?. Crit. Rev. Oncol. Hematol..

[17] Bosma P.J., Chowdhury J.R., Bakker C., Gantla S., de Boer A., Oostra B.A., Lindhout D., Tytgat G.N., Jansen P.L., Oude Elferink R.P. (1995). The genetic basis of the reduced expression of bilirubin UDP-glucuronosyltransferase 1 in Gilbert’s syndrome. N. Engl. J. Med..

[18] Tukey R.H., Strassburg C.P., Mackenzie P.I. (2002). Pharmacogenomics of human UDP-glucuronosyltransferases and irinotecan toxicity. Mol. Pharmacol..

[19] Monaghan G., McLellan A., McGeehan A., Li Volti S., Mollica F., Salemi I., Din Z., Cassidy A., Hume R., Burchell B. (1999). Gilbert’s syndrome is a contributory factor in prolonged unconjugated hyperbilirubinemia of the newborn. J. Pediatr..

[20] Beutler E., Gelbart T., Demina A. (1998). Racial variability in the UDP-glucuronosyltransferase 1 (UGT1A1) promoter: A balanced polymorphism for regulation of bilirubin metabolism?. Proc. Natl. Acad. Sci. USA.

[21] Akaba K., Kimura T., Sasaki A., Tanabe S., Ikegami T., Hashimoto M., Umeda H., Yoshida H., Umetsu K., Chiba H. (1998). Neonatal hyperbilirubinemia and mutation of the bilirubin uridine diphosphate-glucuronosyltransferase gene: A common missense mutation among Japanese, Koreans and Chinese. Biochem. Mol. Biol. Int..

[22] Maruo Y., Nishizawa K., Sato H., Doida Y., Shimada M. (1999). Association of neonatal hyperbilirubinemia with bilirubin UDP-glucuronosyltransferase polymorphism. Pediatrics.

[23] Yusoff S., Takeuchi A., Ashi C., Tsukada M., Ma’amor N.H., Zilfalil B.A., Yusoff N.M., Nakamura T., Hirai M., Harahap I.S. (2010). A polymorphic mutation, c.-3279T>G, in the UGT1A1 promoter is a risk factor for neonatal jaundice in the Malay population. Pediatr. Res..

[24] Sugatani J., Yamakawa K., Yoshinari K., Machida T., Takagi H., Mori M., Kakizaki S., Sueyoshi T., Negishi M., Miwa M. (2002). Identification of a defect in the UGT1A1 gene promoter and its association with hyperbilirubinemia. Biochem. Biophys. Res. Commun..

[25] Amandito R., Rohsiswatmo R., Carolina E., Maulida R., Kresnawati W., Malik A. (2019). Profiling of UGT1A1(*)6, UGT1A1(*)60, UGT1A1(*)93, and UGT1A1(*)28 Polymorphisms in Indonesian Neonates With Hyperbilirubinemia Using Multiplex PCR Sequencing. Front. Pediatr..

[26] Gammal R.S., Court M.H., Haidar C.E., Iwuchukwu O.F., Gaur A.H., Alvarellos M., Guillemette C., Lennox J.L., Whirl-Carrillo M., Brummel S.S. (2016). Clinical Pharmacogenetics Implementation Consortium (CPIC) Guideline for UGT1A1 and Atazanavir Prescribing. Clin. Pharmacol. Ther..

[27] Lv X., Xia Y., Finel M., Wu J., Ge G., Yang L. (2019). Recent progress and challenges in screening and characterization of UGT1A1 inhibitors. Acta Pharm. Sin. B.

[28] de Man F.M., Goey A.K.L., van Schaik R.H.N., Mathijssen R.H.J., Bins S. (2018). Individualization of Irinotecan Treatment: A Review of Pharmacokinetics, Pharmacodynamics, and Pharmacogenetics. Clin. Pharmacokinet..

[29] Kciuk M., Marciniak B., Kontek R. (2020). Irinotecan-Still an Important Player in Cancer Chemotherapy: A Comprehensive Overview. Int. J. Mol. Sci..

[30] Fujita K., Kubota Y., Ishida H., Sasaki Y. (2015). Irinotecan, a key chemotherapeutic drug for metastatic colorectal cancer. World J. Gastroenterol..

[31] Iyer L., King C.D., Whitington P.F., Green M.D., Roy S.K., Tephly T.R., Coffman B.L., Ratain M.J. (1998). Genetic predisposition to the metabolism of irinotecan (CPT-11). Role of uridine diphosphate glucuronosyltransferase isoform 1A1 in the glucuronidation of its active metabolite (SN-38) in human liver microsomes. J. Clin. Investig..

[32] Owens D., Evans J. (1975). Population studies on Gilbert’s syndrome. J. Med. Genet..

[33] Fukuda M., Okumura M., Iwakiri T., Arimori K., Honda T., Kobayashi K., Senju H., Takemoto S., Ikeda T., Yamaguchi H. (2018). Relationship between UGT1A1*27 and UGT1A1*7 polymorphisms and irinotecan-related toxicities in patients with lung cancer. Thorac. Cancer.

[34] Frampton J.E. (2020). Liposomal Irinotecan: A Review in Metastatic Pancreatic Adenocarcinoma. Drugs.

[35] Kalra A.V., Kim J., Klinz S.G., Paz N., Cain J., Drummond D.C., Nielsen U.B., Fitzgerald J.B. (2014). Preclinical activity of nanoliposomal irinotecan is governed by tumor deposition and intratumor prodrug conversion. Cancer Res..

[36] Bardia A., Hurvitz S.A., Tolaney S.M., Loirat D., Punie K., Oliveira M., Brufsky A., Sardesai S.D., Kalinsky K., Zelnak A.B. (2021). Sacituzumab Govitecan in Metastatic Triple-Negative Breast Cancer. N. Engl. J. Med..

[37] Rugo H.S., Bardia A., Marme F., Cortes J., Schmid P., Loirat D., Tredan O., Ciruelos E., Dalenc F., Gomez Pardo P. (2023). Overall survival with sacituzumab govitecan in hormone receptor-positive and human epidermal growth factor receptor 2-negative metastatic breast cancer (TROPiCS-02): A randomised, open-label, multicentre, phase 3 trial. Lancet.

[38] Syed Y.Y. (2020). Sacituzumab Govitecan: First Approval. Drugs.

[39] Hafeez U., Parakh S., Gan H.K., Scott A.M. (2020). Antibody-Drug Conjugates for Cancer Therapy. Molecules.

[40] Michaleas S., Moreno Oliver A., Mueller-Berghaus J., Sarac S.B., van der Elst M.E., Muller-Egert S., Zander H., Enzmann H., Pignatti F. (2022). The European Medicines Agency review of sacituzumab govitecan for the treatment of triple-negative breast cancer. ESMO Open.

[41] Pavone G., Motta L., Martorana F., Motta G., Vigneri P. (2021). A New Kid on the Block: Sacituzumab Govitecan for the Treatment of Breast Cancer and Other Solid Tumors. Molecules.

[42] Fleming P.J., Karpio S., Lombardo N. (2021). Sacituzumab Govitecan for Treatment of Refractory Triple-Negative Metastatic Breast Cancer. J. Adv. Pract. Oncol..

[43] Goldenberg D.M., Cardillo T.M., Govindan S.V., Rossi E.A., Sharkey R.M. (2015). Trop-2 is a novel target for solid cancer therapy with sacituzumab govitecan (IMMU-132), an antibody-drug conjugate (ADC). Oncotarget.

[44] Spring L.M., Nakajima E., Hutchinson J., Viscosi E., Blouin G., Weekes C., Rugo H., Moy B., Bardia A. (2021). Sacituzumab Govitecan for Metastatic Triple-Negative Breast Cancer: Clinical Overview and Management of Potential Toxicities. Oncologist.

[45] Hande K.R. (1998). Etoposide: Four decades of development of a topoisomerase II inhibitor. Eur. J. Cancer.

[46] Watanabe Y., Nakajima M., Ohashi N., Kume T., Yokoi T. (2003). Glucuronidation of etoposide in human liver microsomes is specifically catalyzed by UDP-glucuronosyltransferase 1A1. Drug Metab. Dispos..

[47] Kelly R.J., Rixe O. (2009). Axitinib--a selective inhibitor of the vascular endothelial growth factor (VEGF) receptor. Target. Oncol..

[48] Chen Y., Tortorici M.A., Garrett M., Hee B., Klamerus K.J., Pithavala Y.K. (2013). Clinical pharmacology of axitinib. Clin. Pharmacokinet..

[49] Kelly R.J., Rixe O. (2010). Axitinib (AG-013736). Small Molecules in Oncology.

[50] Sawas A., Radeski D., O’Connor O.A. (2015). Belinostat in patients with refractory or relapsed peripheral T-cell lymphoma: A perspective review. Ther. Adv. Hematol..

[51] Goey A.K., Figg W.D. (2016). UGT genotyping in belinostat dosing. Pharmacol. Res..

[52] Peer C.J., Goey A.K., Sissung T.M., Erlich S., Lee M.J., Tomita Y., Trepel J.B., Piekarz R., Balasubramaniam S., Bates S.E. (2016). UGT1A1 genotype-dependent dose adjustment of belinostat in patients with advanced cancers using population pharmacokinetic modeling and simulation. J. Clin. Pharmacol..

[53] Wang L., Chan C.E.L., Wong A.L., Wong F.C., Lim S.W., Chinnathambi A., Alharbi S.A., Lee L.S., Soo R., Yong W.P. (2017). Combined use of irinotecan with histone deacetylase inhibitor belinostat could cause severe toxicity by inhibiting SN-38 glucuronidation via UGT1A1. Oncotarget.

[54] Goey A.K., Sissung T.M., Peer C.J., Trepel J.B., Lee M.J., Tomita Y., Ehrlich S., Bryla C., Balasubramaniam S., Piekarz R. (2016). Effects of UGT1A1 genotype on the pharmacokinetics, pharmacodynamics, and toxicities of belinostat administered by 48-hour continuous infusion in patients with cancer. J. Clin. Pharmacol..

[55] Reizine N.M., O’Donnell P.H. (2022). Modern developments in germline pharmacogenomics for oncology prescribing. CA Cancer J. Clin..

[56] Zhang N., Liu Y., Jeong H. (2015). Drug-Drug Interaction Potentials of Tyrosine Kinase Inhibitors via Inhibition of UDP-Glucuronosyltransferases. Sci. Rep..

[57] Voigtlaender M., Schneider-Merck T., Trepel M. (2018). Lapatinib. Small Molecules in Oncology.

[58] Midgley R.S., Kerr D.J., Flaherty K.T., Stevenson J.P., Pratap S.E., Koch K.M., Smith D.A., Versola M., Fleming R.A., Ward C. (2007). A phase I and pharmacokinetic study of lapatinib in combination with infusional 5-fluorouracil, leucovorin and irinotecan. Ann. Oncol..

[59] Singer J.B., Shou Y., Giles F., Kantarjian H.M., Hsu Y., Robeva A.S., Rae P., Weitzman A., Meyer J.M., Dugan M. (2007). UGT1A1 promoter polymorphism increases risk of nilotinib-induced hyperbilirubinemia. Leukemia.

[60] Miyamoto S., Kakutani S., Sato Y., Hanashi A., Kinoshita Y., Ishikawa A. (2018). Drug review: Pazopanib. Jpn. J. Clin. Oncol..

[61] Xu C.F., Reck B.H., Xue Z., Huang L., Baker K.L., Chen M., Chen E.P., Ellens H.E., Mooser V.E., Cardon L.R. (2010). Pazopanib-induced hyperbilirubinemia is associated with Gilbert’s syndrome UGT1A1 polymorphism. Br. J. Cancer.

[62] Hahn O., Stadler W. (2006). Sorafenib. Curr. Opin. Oncol..

[63] Mross K., Steinbild S., Baas F., Gmehling D., Radtke M., Voliotis D., Brendel E., Christensen O., Unger C. (2007). Results from an in vitro and a clinical/pharmacological phase I study with the combination irinotecan and sorafenib. Eur. J. Cancer.

[64] Liu Y., Ramirez J., House L., Ratain M.J. (2010). Comparison of the drug-drug interactions potential of erlotinib and gefitinib via inhibition of UDP-glucuronosyltransferases. Drug Metab. Dispos..

[65] Motzer R.J., Johnson T., Choueiri T.K., Deen K.C., Xue Z., Pandite L.N., Carpenter C., Xu C.F. (2013). Hyperbilirubinemia in pazopanib- or sunitinib-treated patients in COMPARZ is associated with UGT1A1 polymorphisms. Ann. Oncol..

[66] Du Z., Wang G., Cao Y.F., Hu C.M., Yang K., Liu Y.Z., Zhang C.Z., Zhang W.H., Zhu Z.T., Sun H.Z. (2018). Everolimus-inhibited multiple isoforms of UDP-glucuronosyltransferases (UGTs). Xenobiotica.

[67] Kweekel D.M., Gelderblom H., Van der Straaten T., Antonini N.F., Punt C.J., Guchelaar H.J. (2008). UGT1A1*28 genotype and irinotecan dosage in patients with metastatic colorectal cancer: A Dutch Colorectal Cancer Group study. Br. J. Cancer.

[68] Tejpar S., Yan P., Piessevaux H., Dietrich D., Brauchli P., Klingbiel D., Fiocca R., Delorenzi M., Bosman F., Roth A.D. (2018). Clinical and pharmacogenetic determinants of 5-fluorouracyl/leucovorin/irinotecan toxicity: Results of the PETACC-3 trial. Eur. J. Cancer.

[69] Shulman K., Cohen I., Barnett-Griness O., Kuten A., Gruber S.B., Lejbkowicz F., Rennert G. (2011). Clinical implications of UGT1A1*28 genotype testing in colorectal cancer patients. Cancer.

[70] Hoskins J.M., Goldberg R.M., Qu P., Ibrahim J.G., McLeod H.L. (2007). UGT1A1*28 genotype and irinotecan-induced neutropenia: Dose matters. J. Natl. Cancer Inst..

[71] Yu Q., Zhang T., Xie C., Qiu H., Liu B., Huang L., Peng P., Feng J., Chen J., Zang A. (2018). UGT1A polymorphisms associated with worse outcome in colorectal cancer patients treated with irinotecan-based chemotherapy. Cancer Chemother. Pharmacol..

[72] Reizine N., Vokes E.E., Liu P., Truong T.M., Nanda R., Fleming G.F., Catenacci D.V.T., Pearson A.T., Parsad S., Danahey K. (2020). Implementation of pharmacogenomic testing in oncology care (PhOCus): Study protocol of a pragmatic, randomized clinical trial. Ther. Adv. Med. Oncol..

[73] Tsai H.L., Huang C.W., Lin Y.W., Wang J.H., Wu C.C., Sung Y.C., Chen T.L., Wang H.M., Tang H.C., Chen J.B. (2020). Determination of the UGT1A1 polymorphism as guidance for irinotecan dose escalation in metastatic colorectal cancer treated with first-line bevacizumab and FOLFIRI (PURE FIST). Eur. J. Cancer.

[74] Harada K., Yamamura T., Muto O., Nakamura M., Sogabe S., Sawada K., Nakano S., Yagisawa M., Muranaka T., Dazai M. (2023). Correlation of UGT1A1 Gene Polymorphisms or Prior Irinotecan Treatment and Treatment Outcomes of Nanoliposomal-Irinotecan plus 5-Fluorouracil/Leucovorin for Pancreatic Ductal Adenocarcinoma: A Multicenter, Retrospective Cohort Study (HGCSG2101). J. Clin. Med..

[75] Su Y.Y., Chiang N.J., Chang J.S., Wang Y.W., Shen B.N., Li Y.J., Hwang D.Y., Shan Y.S., Chen L.T. (2023). The association between UGT1A1 polymorphisms and treatment toxicities of liposomal irinotecan. ESMO Open.

[76] Karas S., Innocenti F. (2022). All You Need to Know About UGT1A1 Genetic Testing for Patients Treated With Irinotecan: A Practitioner-Friendly Guide. JCO Oncol. Pract..

[77] Rugo H.S., Tolaney S.M., Loirat D., Punie K., Bardia A., Hurvitz S.A., O’Shaughnessy J., Cortes J., Dieras V., Carey L.A. (2022). Safety analyses from the phase 3 ASCENT trial of sacituzumab govitecan in metastatic triple-negative breast cancer. NPJ Breast Cancer.

[78] Bardia A., Messersmith W.A., Kio E.A., Berlin J.D., Vahdat L., Masters G.A., Moroose R., Santin A.D., Kalinsky K., Picozzi V. (2021). Sacituzumab govitecan, a Trop-2-directed antibody-drug conjugate, for patients with epithelial cancer: Final safety and efficacy results from the phase I/II IMMU-132-01 basket trial. Ann. Oncol..

[79] Anai S., Iwama E., Yoneshima Y., Otsubo K., Tanaka K., Nakanishi Y., Okamoto I. (2018). Association of nephrotoxicity during platinum-etoposide doublet therapy with UGT1A1 polymorphisms in small cell lung cancer patients. Lung Cancer.

[80] Negoro Y., Yano R., Yoshimura M., Suehiro Y., Yamashita S., Kodawara T., Watanabe K., Tsukamoto H., Nakamura T., Kadowaki M. (2019). Influence of UGT1A1 polymorphism on etoposide plus platinum-induced neutropenia in Japanese patients with small-cell lung cancer. Int. J. Clin. Oncol..

[81] Narita S., Igarashi R., Tsuchiya N., Inoue T., Fujiyama N., Numakura K., Tsuruta H., Kagaya H., Saito M., Niioka T. (2017). The impact of UGT1A1 and SLCO1B1 genetic polymorphisms and pharmacokinetics of axitinib on clinical safety and efficacy in patients with metastatic renal cell carcinoma. J. Clin. Oncol..

[82] Spraggs C., Budde L., Briley L., Bing N., Newstat B., Rappold E., Mooser V., Cardon L., Stein S. (2009). Hyperbilirubinemia in Lapatinib Treated Patients Is Associated with Gilbert’s Syndrome UGT1A1 Polymorphism. Cancer Res..

[83] Shibata T., Minami Y., Mitsuma A., Morita S., Inada-Inoue M., Oguri T., Shimokata T., Sugishita M., Naoe T., Ando Y. (2014). Association between severe toxicity of nilotinib and UGT1A1 polymorphisms in Japanese patients with chronic myelogenous leukemia. Int. J. Clin. Oncol..

[84] Ivanyi P., Eggers H., Hornig M., Kasper B., Heissner K., Kopp H.G., Kirstein M., Ganser A., Grunwald V. (2020). Hepatic toxicity during regorafenib treatment in patients with metastatic gastrointestinal stromal tumors. Mol. Clin. Oncol..

[85] Saif M.W., Smith M.H., Maloney A., Diasio R.B. (2016). Imatinib-induced hyperbilirubinemia with UGT1A1 (*28) promoter polymorphism: First case series in patients with gastrointestinal stromal tumor. Ann. Gastroenterol..

[86] Liu Y., Ramirez J., House L., Ratain M.J. (2010). The UGT1A1*28 polymorphism correlates with erlotinib’s effect on SN-38 glucuronidation. Eur. J. Cancer.

[87] Cheng X., Lv X., Qu H., Li D., Hu M., Guo W., Ge G., Dong R. (2017). Comparison of the inhibition potentials of icotinib and erlotinib against human UDP-glucuronosyltransferase 1A1. Acta Pharm. Sin. B.

[88] https://www.aiom.it/raccomandazioni-2019-per-analisi-farmacogenetiche/.

[89] Relling M.V., Klein T.E. (2011). CPIC: Clinical Pharmacogenetics Implementation Consortium of the Pharmacogenemocis Research Network. Clin. Pharmacol. Ther..

[90] https://www.pharmgkb.org/guidelineAnnotation/PA166104951.

[91] Ehmann F., Caneva L., Papaluca M. (2014). European Medicines Agency initiatives and perspectives on pharmacogenomics. Br. J. Clin. Pharmacol..

[92] https://www.accessdata.fda.gov/drugsatfda_docs/label/2022/020571Orig1s053lbl.pdf.

[93] https://www.accessdata.fda.gov/drugsatfda_docs/label/2020/206256s003lbl.pdf.

[94] https://www.fda.gov/medical-devices/precision-medicine/table-pharmacogenetic-associations.

[95] https://www.nccn.org/professionals/physician_gls/pdf/colon.pdf.

[96] Etienne-Grimaldi M.C., Boyer J.C., Thomas F., Quaranta S., Picard N., Loriot M.A., Narjoz C., Poncet D., Gagnieu M.C., Ged C. (2015). UGT1A1 genotype and irinotecan therapy: General review and implementation in routine practice. Fundam. Clin. Pharmacol..

